# Advances in Physicochemical and Biochemical Characterization of Archaeosomes from Polar Lipids of *Aeropyrum pernix* K1 and Stability in Biological Systems

**DOI:** 10.1021/acsomega.2c07406

**Published:** 2023-01-13

**Authors:** Jan Kejžar, Ilja Gasan Osojnik Črnivec, Nataša Poklar Ulrih

**Affiliations:** † Department of Food Science and Technology, Biotechnical Faculty, University of Ljubljana, Jamnikarjeva 101, SI-1000 Ljubljana, Slovenia; ‡ The Centre of Excellence for Integrated Approaches in Chemistry and Biology of Proteins (CipKeBiP), Jamova 39, SI-1000 Ljubljana, Slovenia

## Abstract

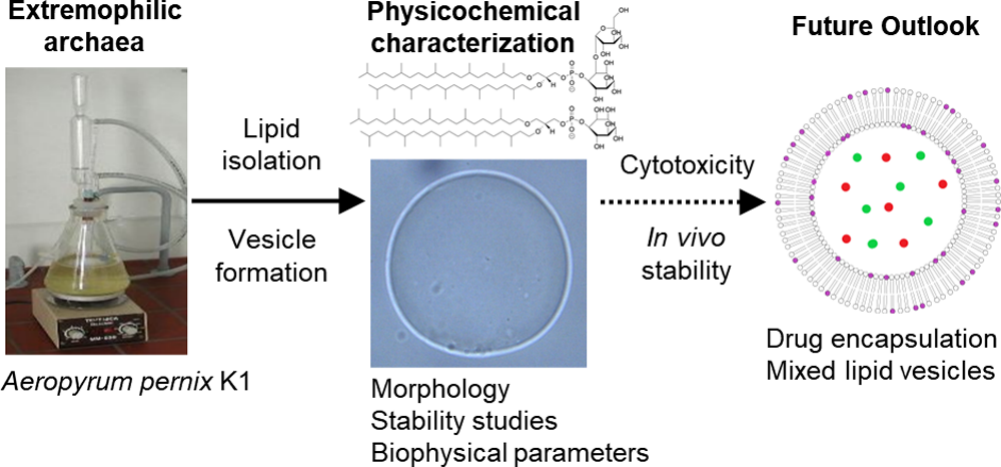

Archaeosomes are vesicles made from archaeal lipids. They are characterized by remarkable thermostability, resistance to enzymatic degradation, long-term stability, and immunomodulatory properties. In this review the current status of physicochemical properties of archaeal lipids and their stability in biological systems is presented, focusing on total polar lipids from *Aeropyrum pernix* K1. The isolated total polar lipids from *Aeropyrum pernix* K1 consist exclusively of glycerol ether lipids with isoprenoid groups attached to glycerol via ether linkages. More specifically, the two major polar lipids extracted from the membranes are C_25,25_-achaetidyl(glucosyl)inositol and C_25,25_-achaetidylinositol. An overview of the results of the effects of temperature and pH on the stability, structural organization, fluidity, and permeability of archaeosomes composed of pure C_25,25_ was examined by a combination of techniques, including fluorescence emission spectroscopy, electron paramagnetic resonance, differential scanning calorimetry, and confocal microscopy. We also compared the physicochemical properties of pure vesicles composed of either archaeal lipids or conventional lipids (e.g., 1,2-dipalmitoyl-*sn*-glycero-3-phosphocholine) with mixed vesicles composed of both lipid types. Archaeal lipids are discussed in terms of their potential use as a targeted drug delivery system based on the results of *in vivo* and cytotoxicity studies.

## Introduction

Archaea were not officially recognized as a third domain of life until 30 years ago, when a reclassification based on phylogenetic analysis of the rRNA sequence was proposed.^1^ Archaea are a unique group of organisms that are immediately recognizable as “extremophiles” because their species are widely distributed in inhospitable environments (e.g., hot acid springs and undersea volcanic fields) and hold numerous records for growth and survival in extreme environments^2^ such as high salinity, very low or very high pH and temperature, high pressure, and low oxygen concentration. One of the main factors that enable the remarkable growth of Archaea in extreme environments is the high integrity of their cell envelope, which is largely due to their unique cell membrane lipids. Unlike conventional lipids, which consist of ester-linked fatty acids attached to glycerol-3-phosphate, archaeal lipids are ether-linked isoprenoid chains with a glycerol-1-phosphate backbone. Additionally, the different stereochemistry of glycerol makes archaeal lipids resistant to enzymatic degradation. Early on, the postulated excellent stability of archaeal lipids sparked interests of studying archaeal lipids to produce vesicles (archaeosomes) that should in theory possess similar characteristics of archaeal cells. Vesicles are self-assembled bilayer spheres of amphipathic molecules, in which the hydrophilic head groups face the outer aqueous medium and the hydrocarbon chains assemble toward the hydrophobic interior.^3^ Their biocompatibility and amphiphilic nature could make them ideal drug delivery systems as they can entrap both hydrophilic and hydrophobic molecules. However, achieving improved drug encapsulation, long-term storage, and resistance, as well as fine-tuned release and liposome pharmacokinetics and pharmacodynamics, requires maintaining specific lipid chemistry (types of lipids and ratios between them) at highly controlled vesicle preparation parameters. Furthermore, despite vast research done on all liposomal formulations and their applications into drug delivery systems, there are only around 20 liposome-based delivery systems registered by the U.S. Food and Drug Administration.^4^ Most of them are liposomal formulations for intravenous use.

It is difficult to compare the results found in the literature on archaeosomes because the different archaeal species live in very different niche conditions, which is also reflected in the molecular structure of their lipids and consequently in the properties of their membrane. In addition, studies on archaeosomes are highly specialized and often deal with an extension of different characterization techniques, often due to the specificity of the studies and working materials, typically using nonstandard extraction procedures and characterization methods that are precisely tailored to the original biological samples. Thus, archaeosomes made from lipids of different archaea, as well as archaeosomes made from different lipid fractions of the same archaeal origin, may have unique properties, and it is critical to examine the lipids of specific archaea and to be cautious when referring to archaeal lipids without mentioning the name of the specific archaeon. In our review, we therefore focus on the lipids of *A. pernix* K1 and their physicochemical characterization while also examining their stability in biological systems. In addition, the review provides an overview of studies focusing on the admixture of archaeal lipids to conventional ester lipid mixtures to improve the stability and feasibility of various liposomal formulations.

## Stability of Heterochiral Mixed Membranes Made from Archaeal *sn*-Glycerol-1-phosphate-Type Lipids and Conventional Ester *sn*-Glycerol-3-phosphate-Type Lipids

Archaeal membrane lipids possess *sn*-G1P chirality of the glycerol backbone, which differs from *sn*-G3P chirality of eukaryotic and bacterial membrane lipids. Heterochiral membranes composed of both *sn*-G3P and *sn*-G1P type lipids were initially expected to be thermodynamically unstable and to eventually segregate due to lipid incompatibilities.^5^ By using nearest-neighbor analysis, Uragami et al.^5^ observed a large influence of glycerol backbone chirality on the lipid mixing of analogous lipids. Additionally, Nassoy et al.^6^ have shown that racemic mixtures of d- and l-myristoylalanine are indeed unstable in monolayers as they undergo chiral discrimination and consequently chiral segregation into d- and l-domains in approximately 1 h.^6^ However, reported segregation versus stable bilayers might be due to the structural difference between amino acid lipids, (myristoylalanine), and glycerol backbone lipids used on contrasting studies.^7−9^ In contrast, studies performed on mixed membranes composed from archaeal lipids and phosphatidylcholine have reported better thermodynamic stability relative to pure phosphatidylcholine vesicles and discussed suitability for drug delivery systems, indicating a promising strategy for membrane studies. Furthermore, Fan et al.^9^ made mixed vesicles from egg phosphatidylcholine and polar lipids isolated from the archaeon *Sulfolobus solfataricus* at a 2:1 molar ratio which had lower leakage of calcein in the presence of destabilizing agents (Ca^2+^ and polyethylene glycol) than pure vesicles made from either polar lipids or egg phosphatidylcholine. In contrast, Sprott et al.^10^ reported difficulties in producing vesicles from a mixture of egg phosphatidylcholine and the polar lipid extract of *S. solfataricus* as they observed formation of unwanted lipid aggregates. Discrepancies between studies could be due to variations in purity and composition of the obtained polar lipid extracts. For example, Cavagnetto et al.^11^ were unable to prepare vesicles from polar lipid extract of *S. solfataricus*, possibly due to the packing parameter of lipids, which was higher than one. Shimada et al.^7^ further showed that the heterochiral mixed vesicles composed from 1,2-dipalmitoyl-*sn*-glycero-3-phosphocholine (DPPC) and lipids obtained from *Sulfolobus tokodaii* (archaeal *sn*-G1P type) were thermally stable and had low 5-carboxyfluorescein leakage at high temperatures (80, 100, and 120 °C) which supports previous results.^9^ They also found that mixed vesicles made of DPPC and the main polar lipids (purified fraction of *sn*-G1P lipids isolated from archaeon *Thermoplasma acidophilum*) had lower leakage of 5-carboxyfluorescein compared to either pure DPPC or pure main polar lipids and were considered thermally stable for at least 5 h at 100 °C.

The phase separation in monopolar–bipolar lipid mixtures is driven by a packing mismatch between lipophilic regions of hydrocarbon chains from conventional lipids and membrane-spanning diether lipids from archaea. Interestingly, Gmajner et al.^12^ reported that small unilamellar vesicles composed from mixtures of DPPC and diether polar lipids isolated from archaeon *Aeropyrum pernix* K1 (at 1:3, 1:1, and 3:1 molar ratios) had significantly lower calcein leakage than pure DPPC vesicles at temperatures from 10 to 98 °C. This is presumably due to more favorable interactions of conventional monopolar lipids with the monopolar nature of *A. pernix* lipids in comparison with other archaeal bipolar lipids. Shimada et al.^7^ also observed enhanced thermal stability and low solute leakage of mixed vesicles made from DPPC and monopolar lipids from either *A. pernix* (*sn*-G1P) or the extreme thermophilic bacteria *Thermus thermophilus* (*sn*-G3P monopolar lipids). Contrasting results from other studies^13,14^ showing decreased thermal stability and higher solute leakage could be due to the origin of the monopolar lipids which were not isolated from thermophiles, while the monopolar lipids used by Gmajner et al.^8^ and Shimada et al.^7^ were from thermophiles *A. pernix* and *T. thermophilus*, respectively. It seems the chirality of the monopolar lipids is not a general predictor of successful mixing and vesicle formation of monopolar lipids as both homochiral and heterochiral mixtures were found to form vesicles. Furthermore, successful mixing of monopolar lipids from thermophilic archaea and bacteria (*A. pernix* and *T. thermus*, respectively) might be possible due to the unique molecular structure of monopolar lipids such as sugar moieties on the main polar head and relatively large isoprenoidic chain.

The results of the mentioned studies show that heterochiral mixed vesicles made from lipids isolated from thermophilic archaea generally have low solute leakage in a wide temperature range. Additionally, Shimada et al.^7^ showed a significant effect of the length of hydrocarbon chains in phosphatidylcholine-type lipids on the thermal stability and solute leakage of mixed membranes even in the absence of chiral segregation. They found that the 5-carboxyfluorescein leakage of the mixed membranes is lowest when mixtures are composed from similar chain length lipids. Accordingly, the main polar lipids from *T. acidophilum* are C_40_ caldarchaeol lipids, and their leakage at high temperatures (80, 100, and 120 °C) was reported to be the lowest in mixtures at a 2:1 molar ratio with DPPC (C_16_), 1,2-distearoyl-*sn*-glycero-3-phosphocholine (C_18_), and 1,2-diarachidoyl-*sn*-glycero-3-phosphocholine (C_20_). In contrast, the 5-carboxyfluorescein leakage was drastically increased in mixtures with 1,2-dilauroyl-*sn*-glycero-3-phosphocholine (C_12_), 1,2-dimyristoyl-*sn*-glycero-3-phosphocholine (C_14_), and 1,2-dibehenoyl-*sn*-glycero-3-phosphocholine (C_22_) lipids. Therefore, the thermal stability and leakage of mixed membranes is better when the sum length of two monopolar lipid carbon chains is similar to the length of the bipolar caldarchaeol molecule. Studies seem to conclude that molecular packing is more important than the chirality of the glycerol backbone which is not crucial for the integrity of the membrane. Discrepancies between the results of some studies could be caused by variations in lipid structure and their ratios due to the natural origin of archaeal lipids. Further research in lipid analysis and the structure–function relationship is required in this field, as several studies in the past have not been focused on detailed lipid characterization when studying the stability of membranes.

## Lipids from Thermophilic Archaeon *Aeropyrum pernix* K1

Archaeon *Aeropyrum pernix* K1 was first isolated from a marine underwater hydrothermal vent at the vicinity of the Kodakara Island, Japan.^15^ It was the first discovered obligate aerobic hyper thermophilic organism with the ability to grow at temperatures up to 100 °C. It grows optimally in protein-rich media with temperatures at 90–95 °C, pH 7.0, and 3.5% salinity.

**Figure 1:**
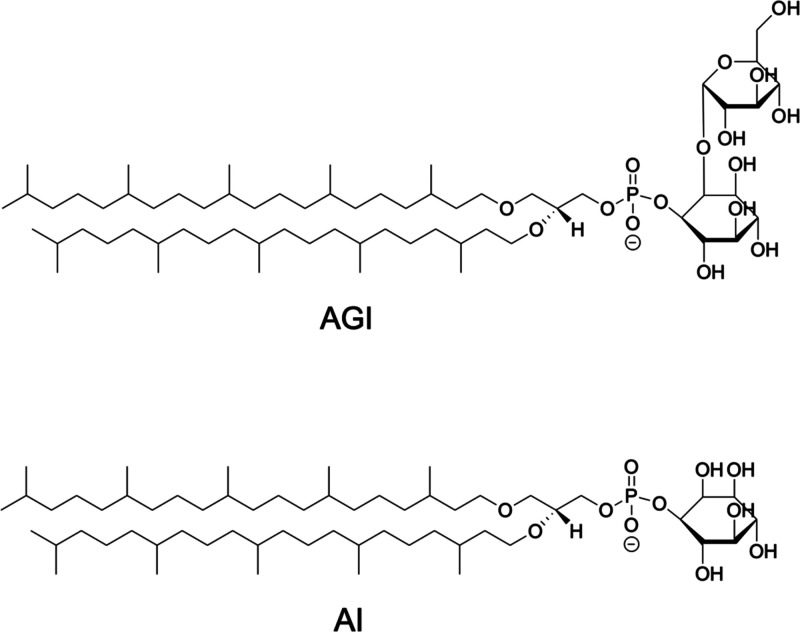
Structural formulas of 2,3-di-*O*-sesterterpanyl-*sn*-glycerol-1-phospho-1′-(2′-*O*-α-d-glucosyl)-myo-inositol (C_25,25_-archetidyl(glucosyl)inositol) (AGI) and 2,3-di-*O*-sesterterpanyl-*sn*-glycerol-1-phospho-myo-inositol (C_25,25_-archetidylinositol) (AI).

Isolated polar lipids from hyperthermophile archaeon *A. pernix* K1 consist of two fractions, with predominantly 2,3-di-*O*-sesterterpanyl-*sn*-glycerol-1-phospho-1′-(2′-O-α-d-glucosyl)-myo-inositol (C_25,25_-archaetidyl(glucosyl)inositol) at about 91 mol % and 2,3-di-*O*-sesterterpanyl-*sn*-glycerol-1-phospho-myo-inositol (C_25,25_-archaetidylinositol) at 9 mol %^16^ (Figure 1). Membranes composed of C_25,25_ diether lipid species are 20% thicker compared to C_20,20_ archaeal lipids. While the C_25_-isoprenoid chain lipids were previously reported for haloalkaliphiles,^17^ they were only present in small amounts with bulk lipids being C_20,25_ and C_20,20_ species. C_25,25_ species are also absent in sulfur-dependent hyper thermophilic archaea as they contain tetraether lipids without sugar moieties.^16^ This stark difference in chemical structure of the membrane lipids suggests that bipolar tetraether lipids are not necessary for thermal adaptation.

Furthermore, in terms of cultivation feasibility and technical complexity that often prevent commercial applications of archaea, *A. pernix* are relatively easy to grow compared to anaerobic archaea species, and their lipid composition is stable and generally not affected by growing conditions.^18,19^ Authors also presume that the batch grow process could be replaced by a continuous one, increasing the yield by approximately 10-fold.^18^

### Characterization of Vesicles Made Solely from *Aeropyrum pernix* K1 Lipids and the Mixture with 1,2-Dipalmitoyl-*sn*-glycero-3-phosphocholine

Morphology, size distribution, and zeta potential of mixed vesicles made from C_25,25_ lipids and DPPC with different mol % of C_25,25_ lipids (0, 25, 50, 75, and 100) were investigated by Gmajner et al.^8^ Thorough characterization was made in this early work on vesicles of different sizes and morphologies using various production methods: small unilamellar vesicles (SUV, sonication method), large unilamellar vesicles (LUV, extrusion method through a 100 nm membrane), and multilamellar vesicles (MLV, thin-film hydration method).

Size distribution and zeta potential were determined by dynamic light scattering (DLS) and phase analysis light scattering (PALS), respectively. Mixed C_25,25_-DPPC MLV had smaller sizes at 530 ± 370 nm to 570 ± 370 nm (for 50 mol % and pure C_25,25_ vesicles, respectively) compared to pure DPPC MLV at an approximate mean diameter of 3 μm. Further increase in the portion of C_25,25_ lipids also considerably lowered zeta potential, decreasing from −7 ± 20 mV for pure DPPC to −94 ± 15 mV for pure C_25,25_ lipids. Mean diameters of pure DPPC LUV and SUV were measured at 190 ± 120 nm and 75 ± 30 nm, respectively. Addition of 25 mol % C_25,25_ lipids decreased the size of LUV and SUV to 110 ± 25 nm and 40 ± 25 nm, with no further decreases in size or zeta potential at higher mol % of C_25,25_ lipids. In all cases, incorporation of 25 mol % C_25,25_ lipids into the zwitterionic DPPC vesicles decreased the zeta potential below −30 mV, i.e., the mark where the colloid suspensions are considered to be stable. In summary, mixed SUV and LUV maintained uniform size distribution and zeta potentials at all molar ratios of C_25,25_ and DPPC. All DLS measurements were repeated after 41 days, and no differences were observed compared to the fresh formulations (day 1 results), except for pure DPPC vesicles which either had marginally increased size due to vesicle fusion (for SUV) or were completely unstable due to lipid aggregation (for LUV).

**Figure 2:**
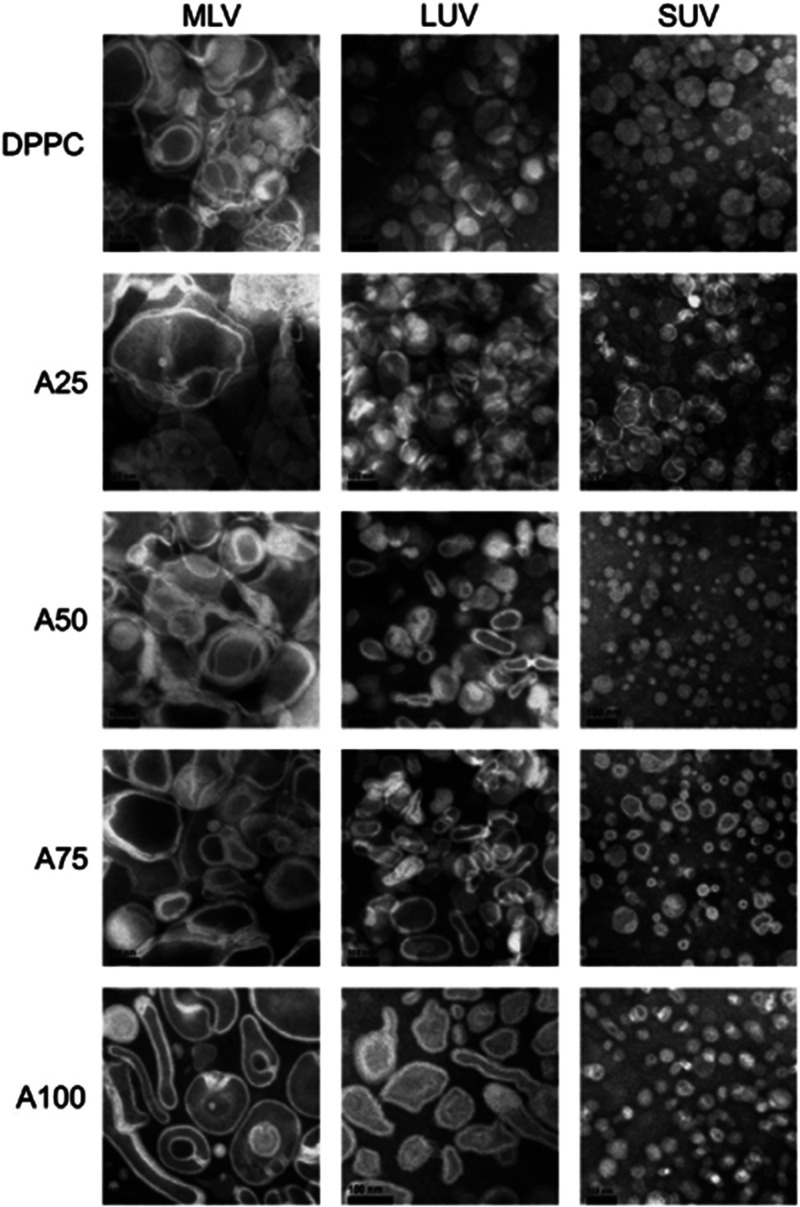
Transmissive electron microscopy images of mixed C_25,25_/DPPC vesicles (MLV, LUV, and SUV) composed of different molar ratios of the archaeal C_25,25_ lipids: 0% (DPPC), 25% (A25), 50% (A50), 75% (A75), and 100% (A100) at pH 7.0. Reprinted with permission from Gmajner, D.; Grabnar, P. A.; Žnidarič, M. T.; Štrus, J.; Šentjurc, M.; Ulrih, N. P. Structural Characterization of Liposomes Made of Diether Archaeal Lipids and Dipalmitoyl-L-α-Phosphatidylcholine. *Biophys. Chem.***2011**, 158 (2–3), 150–156. 10.1016/j.bpc.2011.06.014. Copyright 2011 Elsevier.^8^

Liposome morphology was further examined by transmissive electron microscopy (TEM) using the negative-staining method with ammonium molybdate.^8^Figure 2 represents TEM micrographs of mixed MLV, LUV, and SUV with different ratios of C_25,25_ lipids. Addition of C_25,25_ lipids significantly impacted the morphology of MLV as they were smaller and less multilamellar. The degree of coalescence and fusion decreased with the increase in mol % of C_25,25_ lipids. Pure archaeal lipid vesicles—archaeosomes (A100)—were clearly separated, but they had irregular shapes and very low lamellarity (Figure 2). Mixed lipid LUV were also considerably smaller and extremely monodisperse (110 ± 25 nm diameter; 0.05 PDI) compared to pure DPPC LUV (190 ± 120 nm diameter; 0.43 PDI) and formed nonspherical shapes at 50 mol % C_25,25_ or higher (Figure 2). In contrast to equimolar LUV, mixed SUV assumed a wider size dispersity despite the size-reducing effect of C_25,25_. Furthermore, the TEM examination of SUV and LUV could not confirm lamellarity at any of the observed ratios of C_25,25_ and DPPC (Figure 2).

**Figure 3:**
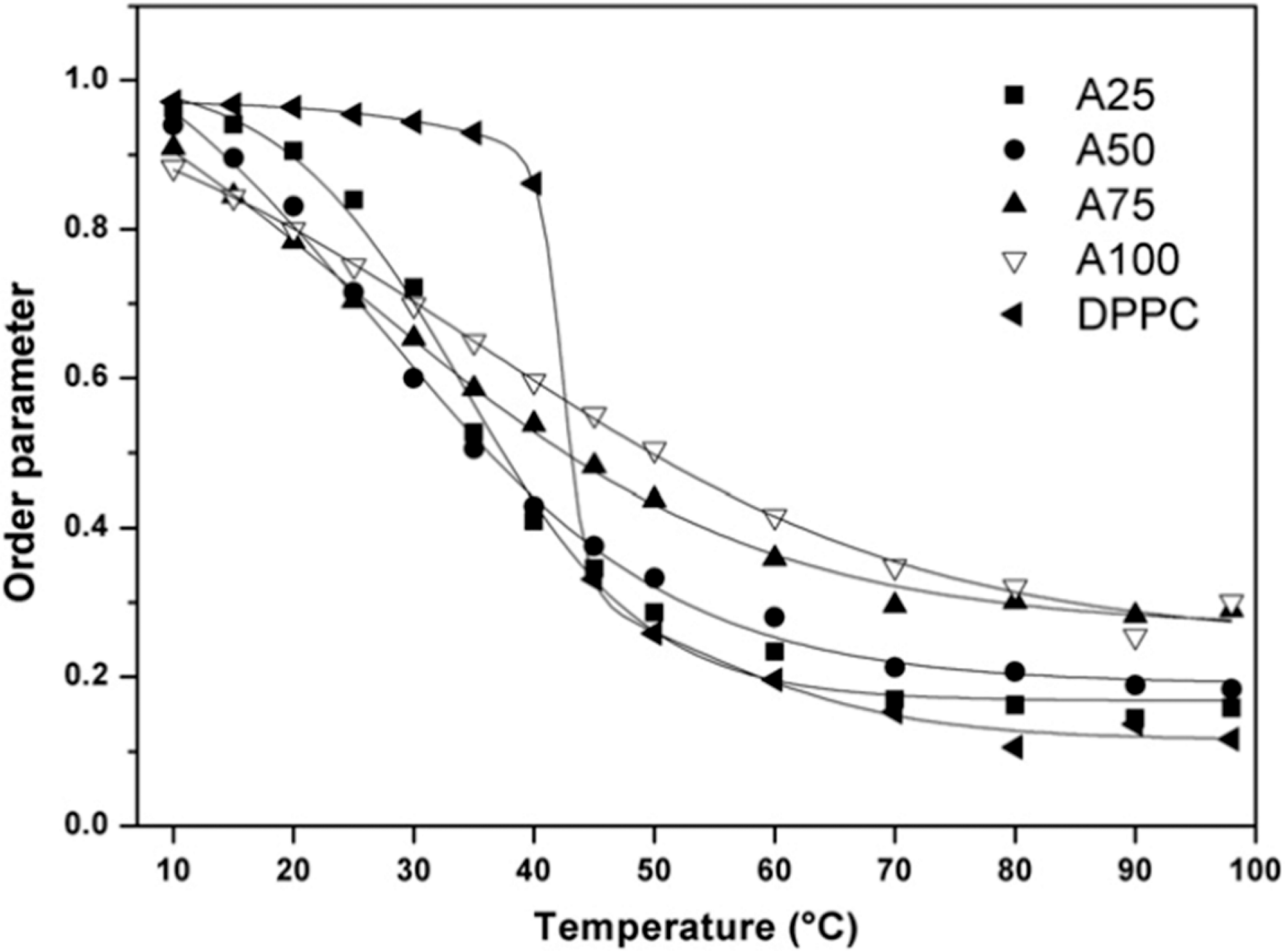
Lipid order parameter of mixed C_25,25_/DPPC vesicles composed of different molar ratios of C_25,25_ lipids at temperatures from 10 to 98 °C: 0% (◀, DPPC), 25% (■, A25), 50% (●, A50), 75% (▲, A75), 100% (▽, A100) at pH 7.0. The solid lines represent nonlinear curve fitting to the data points shown. Reprinted with permission from Gmajner, D.; Grabnar, P. A.; Žnidarič, M. T.; Štrus, J.; Šentjurc, M.; Ulrih, N. P. Structural Characterization of Liposomes Made of Diether Archaeal Lipids and Dipalmitoyl-L-α-Phosphatidylcholine. *Biophys. Chem.***2011**, 158 (2–3), 150–156. 10.1016/j.bpc.2011.06.014. Copyright 2011 Elsevier.^8^

Anisotropy measurements of fluorescent probes are used to study the dynamics of biological membranes,^19^ as their photophysical properties are affected by the changes in the membrane microenvironment in the direct vicinity of the fluorophore. Gmajner et al.^8^ performed anisotropy measurements with a hydrophobic DPH probe at different pH levels and temperatures within SUV archaeosomes composed of C_25,25_ lipids and SUV vesicles made from DPPC. C_25,25_ SUV showed a gradual decrease in anisotropy with increasing temperatures (Figure 3), in the absence of a characteristic transition from the initial ordered to liquid-disordered state as shown for DPPC samples. Values of DPH anisotropy at pH 7.0 (0.249 ± 0.003 at 20 °C) were similar in the alkaline range (pH 7.0 to pH 12.0) but showed a slight increase (0.260 ± 0.005 at pH 4.0) in the acidic range (pH 4.0 to pH 1.0). The anisotropy values of DPPC in gel form (0.337 ± 0.001) were higher than values of C_25,25_ lipids (0.260 ± 0.005) at 20 °C. This infers that archaeosome membranes were in an ordered form or a mixture of one or more states. The absence of the gel to liquid-crystalline phase transition was later confirmed with differential scanning calorimetry in the 0–100 °C range.^12^ Furthermore, the anisotropy values of DPH at 98 °C were higher in C_25,25_-containing archaeosomes compared to pure DPPC vesicles, demonstrating that the C_25,25_ lipids were in more ordered form in relation to pure DPPC.

In mixed vesicles, the phase transition became less apparent with an increasing mol % of the C_25,25_ lipids.^8^ Above the phase transition temperatures of DPPC (41 °C), the addition of C_25,25_ showed a dose-dependent increase of order parameters of mixed vesicles (Figure 3). Regarding the ordering mechanics, the isoprenoid chains of C_25,25_ lipids are presumed to function in the prevention of the pure DPPC from forming highly ordered gel structures (below the DPPC phase transition temperature) and completely disordered structures (above the DPPC phase transition temperature). This has also been observed for other compounds, such as cholesterol, which is known to exhibit similar effects in binary mixtures with DPPC. Due to this reason, combining such compounds could also provide a good strategy and fine-tuning of the membrane characteristics. Indeed, similar effects of combining C_25,25_ and cholesterol in mixtures with DPPC have been described as observed by DSC studies.^20^

Potential variability of membrane lipid composition due to the changes in growing conditions can considerably affect the characteristics of the lipids and, consequently, the repeatability of the studies. Therefore, Ota et al.^19^ performed additional anisotropy value measurements of DPH for C_25,25_ archaeosomes with fractions isolated from *A. pernix* grown at different pH levels (6.0, 7.0, and 8.0). Initial values of the calculated order parameter of DPH from obtained measurements at 20 °C were pH 6.0, 0.72 ± 0.1; pH 7.0, 0.72 ± 0.1; and pH 8.0, 0.73 ± 0.1. No differences were observed in the anisotropy measurements and its respective order parameters of archaeosomes in the tested temperature range, regardless of the growth medium pH level. Additionally, authors’ thin-layer chromatography results showed no visible differences between lipid compositions of *A. pernix* grown at pH levels of 6.0, 7.0, and 8.0.^19^

Electron paramagnetic resonance (EPR) spectra of mixed C_25,25_ and DPPC vesicles were obtained using the MeFASL(10,3) probe at 20 °C and pH 7.0.^8^ Mixed vesicles (at all ratios) had lower empirical correlation times (τ_emp_) than pure DPPC vesicles, indicating that the addition of C_25,25_ lipids made membranes more fluid at temperatures below 40 °C. Above the phase transition temperature (of DPPC), the effect was opposite, as the mixed vesicles were more rigid. Anisotropy results are in good agreement with EPR measurements above 40 °C, while the DPPC vesicles were in the liquid-crystalline state. The contrasting results between EPR and anisotropy measurements above 40 °C could be due to different positions of probes and/or isoprenoid chains of C_25,25_ affecting the deoxyl moiety of MeFASL(10,3) or the DPH fluorophore.^8^

Empirical correlation time (τ_emp_) continuously decreased with increasing temperature (from 10 to 80 °C) for all ratios of mixed vesicles, with a major decline in τ_emp_ observed only with pure DPPC vesicles in the temperature region between 30 and 45 °C. These EPR results indicate the absence of a main phase transition which was also confirmed by anisotropy measurements of DPH and DSC measurements.

**Figure 4:**
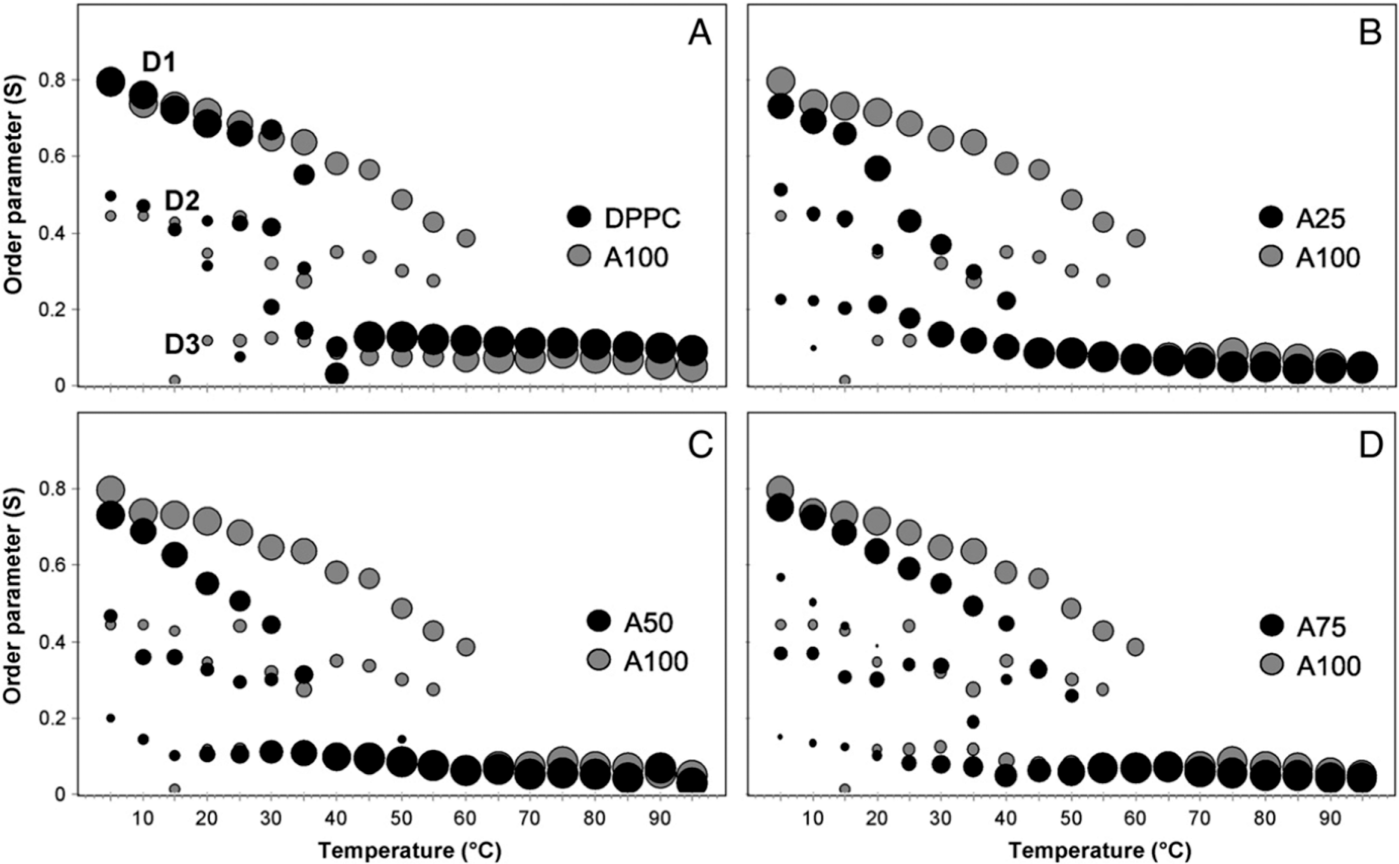
Bubble diagrams of order parameters of mixed C_25,25_/DPPC vesicles in comparison with pure C_25,25_ archaeosomes (A100) composed of different molar ratios of C_25,25_ lipids: (A) 0% (DPPC), (B) 25% (A25), (C) 50% (A50), and (D) 75% (A75) at pH 7.0. Reprinted with permission from Gmajner, D.; Grabnar, P. A.; Žnidarič, M. T.; Štrus, J.; Šentjurc, M.; Ulrih, N. P. Structural Characterization of Liposomes Made of Diether Archaeal Lipids and Dipalmitoyl-L-α-Phosphatidylcholine. *Biophys. Chem.***2011**, 158 (2–3), 150–156. 10.1016/j.bpc.2011.06.014. Copyright 2011 Elsevier.^8^

To further improve the understanding of the structural dynamics of these bilayers and how they change with temperature and different lipid ratios, the authors also performed computer simulations of the EPR spectra. Obtained spectra were the superimposition of the three spectral components with differently shaped lines, which reflected the different modes of the spin-probe motion.^8^ This indicated that C_25,25_ membranes are not homogeneous and are composed of several domains with different fluidity characteristics. At temperatures above 60 °C, the experimental spectra could be described by only one spectral component, indicating the existence of only one domain and, consequently, a homogeneous membrane. Domain types and the changes of the proportions of their respective order parameters are shown as diagrams (bubbles) in Figure 4, where each symbol represents the population of the spin probes and the corresponding nanodomain type.^8^ Pure C_25,25_ archaeosomes were compared to pure DPPC (Figure 4A) and mixed vesicles of different molar ratios of the C_25,25_ and DPPC (Figure 4B–D). D1 is the most ordered nanodomain type, with the order parameter of ∼ 0.75 at 20 °C. D2 represents a less ordered nanodomain with *S* ∼ 0.4 at 20 °C, and D3 is the least-ordered nanodomain type, with *S* ∼ 0.1 across the whole measured temperature range. The order parameter *S* and the proportion of the most ordered domain type D1 decreased with increasing temperature, and at 60 °C only D3 remained, as the properties of the whole membrane were reflected in one motional mode of the spin probe with an *S* order parameter of ∼0.1. Only one spectral component was observed in samples of pure DPPC and 25 mol % C_25,25_ above phase transition temperatures (for DPPC). In mixed vesicles, a proportion of highly ordered domains continuously decreased with increasing temperature, while pure DPPC vesicles exhibited a sudden shift to an exclusively D3 domain (at ∼40 °C) due to a phase transition from the gel-to-liquid crystalline phase. The phase transition was also reflected in the sudden decrease of τ_emp_.

**Figure 5:**
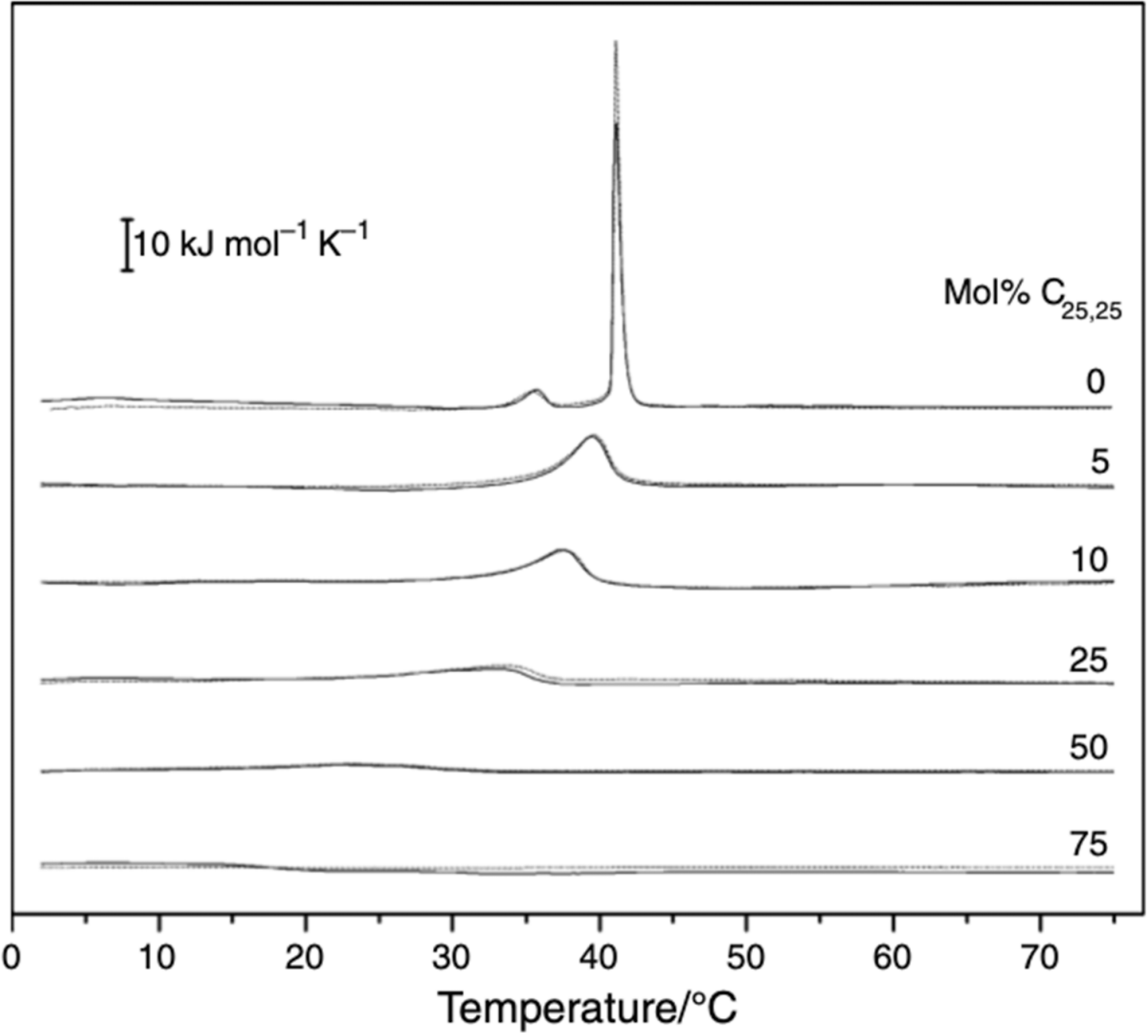
Differential scanning calorimetry curves of mixed C_25,25_/DPPC MLV composed of different molar ratios of C_25,25_ lipids (0%, 5%, 10%, 25%, 50%, and 75%) at pH 7.0. Solid lines indicate the first heating scan, and dashed lines indicate the second heating scan. Reprinted with permission from Gmajner, D.; Ulrih, N. P. Thermotropic Phase Behavior of Mixed Liposomes of Archaeal Diether and Conventional Diester Lipids. *J. Therm. Anal. Calorim.***2011**, 106 (1), 255–260.10.1007/s10973-011-1596-4. Copyright 2011 Springer Nature.^20^

Using differential scanning calorimetry (DSC), Gmajner et al.^20^ then characterized vesicles prepared from isolated total polar lipids of *A. pernix* K1 and have shown the absence of the typical gel-to-liquid crystalline phase transition in the temperature range from 0 to 100 °C. In lieu, however, they observed a gradual broad transition in the temperature range from 0 to 40 °C which coincided with increasing fluidity of the vesicles and its low permeability for entrapped calcein. By DSC, they also studied the thermotropic phase behavior of MLV prepared from mixed C_25,25_/DPPC vesicles containing 0, 5, 25, 75, and 100 mol % C_25,25_ and found that the phase transition of DPPC is significantly affected. Pure DPPC showed two typical endothermic transitions at 35.5 ± 0.3 °C and 41.2 ± 0.3 °C where the latter corresponds to the gel-to-liquid crystalline transition of the lipid side chains (Figure 5). Enthalpy change (Δ*H*) of the DPPC gel-to-liquid crystalline transition was 35.6 ± 0.4 kJ mol^–1^, which agrees with the values found in the literature.^21^ C_25,25_ lipids had a significant effect on the packing of hydrocarbon chains in both gel and liquid crystalline states of DPPC vesicles. At just 5 mol % C_25,25_, the phase transition of C_25,25_ and DPPC mixed vesicles was significantly affected (Figure 5). At higher molar ratios of C_25,25_ (25–75 mol %) in the mixture, the chain-melting transition practically disappeared (Figure 5), indicating that the ordered state was nonexistent in the measured temperature range.

Archaeal lipids have a unique molecular structure which reflects their physicochemical properties including their phase-transition temperatures, which are considerably lower compared to conventional fatty acyl ester lipids,^22^ with some examples of phase transition temperatures being −20 to −15 °C for *Thermoplasma acidophilum* and below −20 °C for diphytanyl (diether) vesicles.^20^ Chong et al.^23^ observed a broad exothermic transition of caldarchaeol archaeosomes made from lipids of *Sulfolobus acidocaldarius* at 78.5 °C with a very small value of Δ*H*.

**Figure 6:**
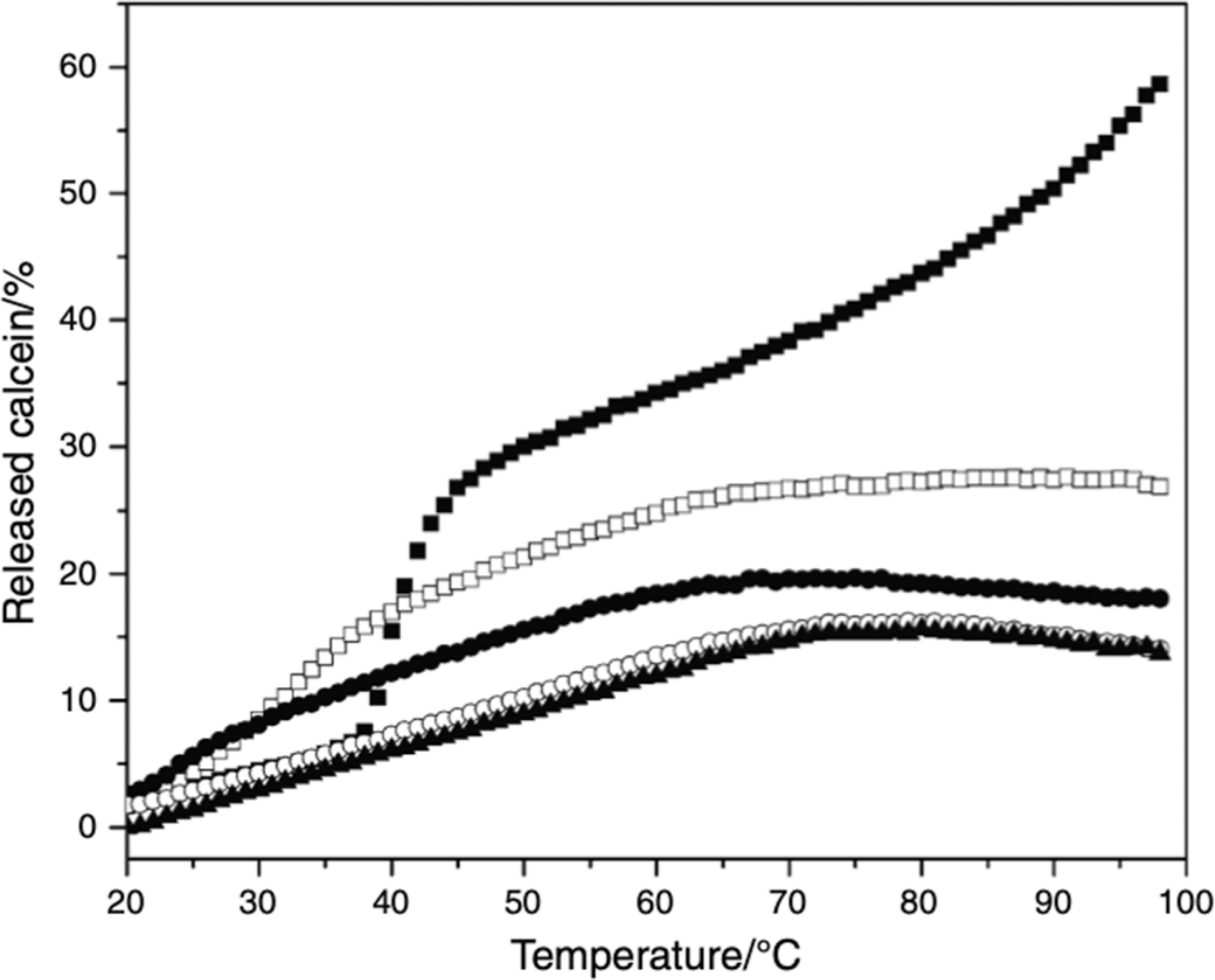
Calcein release of mixed C_25,25_/DPPC vesicles composed of different molar ratios of C_25,25_ lipids at temperatures from 10 to 98 °C: 0 (■), 25 (□), 50 (●), 75 (○), and 100 (▲) at pH 7.0. Reprinted with permission from Gmajner, D.; Ulrih, N. P. Thermotropic Phase Behavior of Mixed Liposomes of Archaeal Diether and Conventional Diester Lipids. *J. Therm. Anal. Calorim.***2011**, 106 (1), 255–260. 10.1007/s10973-011-1596-4. Copyright 2011 Springer Nature.^20^

Archaeal lipid membranes were shown to have characteristically very low permeability to solutes.^22^ Gmajner et al.^20^ have shown that incorporation of C_25,25_ lipids from *A. pernix* into mixed SUV made from conventional ester type lipids (e.g., DPPC) can significantly decrease their permeability to solutes such as calcein. Pure C_25,25_ lipid SUV were less temperature sensitive compared to pure DPPC SUV (Figure 6).^12^ Calcein release decreased with increasing molar ratios of C_25,25_ in mixed vesicles. Similar results were also obtained by Shimada et al.^7^ using 5-carboxyfluorescein as a solute. Pure DPPC vesicles released less calcein at lower temperatures (below phase transition) due to highly ordered gel structures but exhibited sudden calcein release at their gel-to-liquid crystalline phase transition. The coexistence of both phases facilitates the formation of grain-boundary defects in the membrane. The number of interfaces between the gel and liquid phase at the transition temperature greatly affects leakage of the membrane and can be reduced by the addition of cholesterol.^24^ DSC results also show that C_25,25_ lipids affect DPPC similarly to cholesterol by preventing the conventional lipids from forming highly ordered gel structures at low temperatures, which can be seen by the absence of the phase-transition peak.

Many components of the cellular membrane are lost during polar lipid isolation process, some of which can have a significant role in maintaining the stability of the membrane (e.g., *S*-layer proteins). Therefore, using EPR and pyrene fluorescence emission measurements, Ulrih et al.^25^ investigated characteristics of live *A. pernix* cells *in vivo* at different pH levels. They observed changes in the membrane structure with temperature, and they were different for *A. pernix* grown at pH 6.0, pH 7.0, and pH 8.0. Additionally, the results are in contrast with studies on pure archaeosomes composed of their respective lipids. Discrepancies between the measurements done on archaeal cells and archaeosomes are presumably due to the influence of various membrane proteins, which are absent in pure polar lipid archaeosomes.

### *In Vitro* and *in Vivo* Stability of Archaeosomes Prepared from Lipids of *A. pernix*

Archaeosomes composed from lipids of *A. pernix* have a good stability at various temperatures and pH ranges and superior physicochemical characteristics which make them a prospective system for advanced drug delivery. However, such formulations demand minimal cytotoxicity to be viable in applicative forms. Due to the uniqueness of archaeal lipids, even in comparison to different archaeal species, their effect and interactions in biological systems still need to be thoroughly examined, in terms of biological effects of both basic components and vesicles. Previous studies have found that vesicles composed from archaeal lipids are either nontoxic or mildly toxic *in vitro*(26) with some studies showing a significant immune response to certain archaeosomes.^27^

Different biological response aspects of various archaeosomal systems have been examined. Napotnik et al.^26^ investigated *in vitro* cytotoxicity of *A. pernix* archaeosomes on various cell lines, rodent and human, using MTT-dye cytotoxicity assays and confocal microscopy of labeled cells. SUV were prepared from PLMF of *A. pernix* K1 according to Gmajner et al.^8^ with the exception of using isotonic buffer for biological systems. Cytotoxicity and calcein leakage was tested on five cell lines, two rodent (Chinese hamster ovary (CHO) and mouse melanoma cells (B17–F1)) and three human cell lines (epithelial colorectal adenocarcinoma cells (CACO-2), the human liver hepatocellular carcinoma cell line (Hep G2), and primary human umbilical vein endothelial cells hybridized with the A549/8 human lung carcinoma cell line (EA.hy926)).

**Figure 7:**
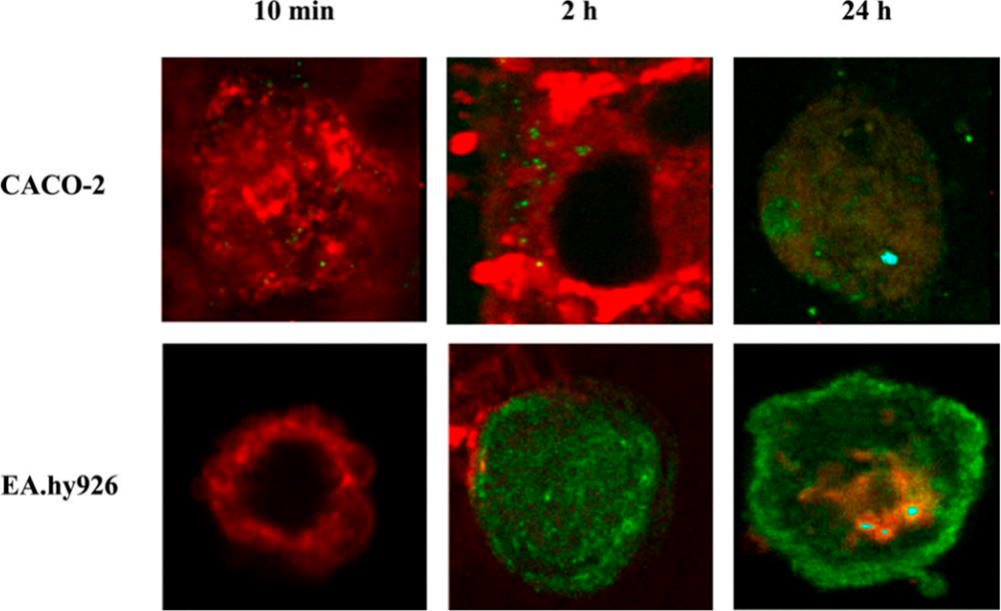
Laser scanning confocal microscope images of cell lines CACO-2 and EA.hy926 incubated with C_25,25_ archaeosomes loaded with calcein (green) at different incubation times. Cells were labeled with Vybrant 1 DiI cell-labeling solution for membranes (red) prior to incubation with archaeosomes. Longitudinal sections are shown. Reprinted with permission from Napotnik, T. B.; Valant, J.; Gmajner, D.; Passamonti, S.; Miklavčič, D.; Ulrih, N. P. Cytotoxicity and Uptake of Archaeosomes Prepared from *Aeropyrum Pernix* Lipids. *Hum. Exp. Toxicol.***2013**, 32 (9), 950–959. 10.1177/0960327113477875. Copyright 2013 SAGE Publications.^26^

Archaeosomes showed no toxicity to human CACO-2 and Hep G2 cells but exhibited a strong toxic effect on the Ea.hy926 cell line with LD50 (medial lethal dose) around 0.8 μg/mL. Toxicity to rodent cell lines was moderate with an LD50 of around 625 μg/mL for B16–F1 and CHO cells. Using CLSM (confocal laser scanning fluorescence microscopy) the authors observed archaeosomes interacting with cells in three distinct phases: (1) adsorption onto the cell surface; (2) transport to the cell interior; and (3) releasing load (calcein in this case) into the cytoplasm.^26^ The duration and intensity of each phase varied between cell types. The attachment to the cell surface and subsequent release of calcein into the cytoplasm were observed after approximately 24 h in B16–F1, CHO, CACO-2, and Hep G2, with the exception of the EA.hy926 cell line where the whole process took only about 30 min. CLSM images also showed intensive green coloring of the cytoplasm in the case of EA.hy926 cells, while in the case of B16–F1, CHO, CACO-2, and Hep G2 cells intact archaeosomes persisted inside the cells even after 24 h (Figure 7). Interestingly, archaeosomes were cytotoxic toward EA.hy926 cells possibly due to fusion of archaeosomes with cells (in contrast to endocytosis), which could disrupt permeability and other critical parameters of EA.hy926 cell membranes.

It should be noted that the majority of conventional liposomal formulations does not exhibit cytotoxicity, with the exception in cases of inclusion of cationic lipids resulting in charge-mediated fusion as well as in the presence of fusogenic 1,2-dioleoyl-*sn*-glycero-3-phosphoethanolamine, which was shown to be highly toxic toward macrophages *in vitro*.^28^

Observed archaeosomes remained intact within the cytoplasm; therefore, the endocytotic pathways in these cases seem to be within the mechanisms of vesicle uptake in observed cell lines and not through lipid fusion. Indeed, *in vitro* studies had shown endocytosis as a consequential mechanism for liposome internalization by the cells.^29^ In more detail, the vesicles are internalized by phagocytosis in phagocytic cells after binding to the surface of the cells, and in nonphagocytic cells the main endocytotic pathway is caveolae-mediated or clathrin-mediated, although vesicles can also enter the cells by macropinocytosis or other pathways. Furthermore, endocytosis of negatively charged archaeosomes is also not unexpected as charged vesicles (with either negative or positive charge) are known to be prone to endocytosis and are internalized faster by endocytotic cells compared to neutral vesicles. It should also be noted that different cells interact differently with vesicles due to the specificity of their respective binding sites on their surface.^26^

Furthermore, the fast release of calcein and high toxicity of archaeosomes observed in EA.hy926 cells were attributed to the higher rate of uptake, fast endocytosis, and apparent fusion of archaeosomes with cell membranes. It is important to understand that EA.hy926 cells express numerous surface molecules characteristic of the human vascular endothelium and are therefore used as an *in vitro* experimental model for studying vascular functions such as adhesion and angiogenesis.^26^ The high rate of uptake of archaeosomes into EA.hy926 cells could therefore be explained by archaeosomes interacting with these specific surface molecules. The resulting cytotoxicity could be due to a combination of archaeal lipids fusing with cell membranes and perturbing membrane states and/or affecting other cell mechanisms. In other cell lines, the majority of archaeosomes stayed intact inside of cells and did not release calcein to the same extent exhibited in EA.hy926 cells. Further research is needed to facilitate drug release from internalized archaeosomes, with techniques such as electroporation using nanosecond electric pulses^30^ or modifications to lipid composition. Further studies on mixed liposomal formulations comprised from conventional and archaeal lipids could provide interesting data, as did Gmajner et al.,^8^ who showed that the physicochemical stability of mixed DPPC/archaeal vesicles is higher compared to pure DPPC vesicles.

Biological instability could prove to be an issue with pure *A. pernix* lipids, as negatively charged vesicles containing anionic lipids are known to be prone to rapid opsonization and uptake by the reticuloendothelial system.^26^ However, with the addition of neutral lipids in the liposomal formulation the negative charge could be lessened while still preserving the colloidal stability effect of zeta potential (below −30 mV). Multilayered structures or matrix incorporation to prevent direct contact with the archaeosomal surfaces might also provide promising strategies.

Rezelj et al.^31^ demonstrated successful incorporation of cholesterol into C_25,25_ lipid membranes, producing mixed lipid vesicles with low calcein permeability. Addition of cholesterol induced increased ordering and tighter lipid packing of the bilayer, ultimately increasing the overall stability of the vesicles. Additionally, successful preparation of cholesterol/C_25,25_ nanodiscs and giant unilamellar vesicles (GUV) was demonstrated as a good model system for studying membrane-interacting proteins, some of which require cholesterol as a crucial component for successful binding and activity (such as perfringolysin O). Furthermore, the authors also showed that although it was postulated that listeriolysin O only interacts with cholesterol-containing membranes^31^ listeriolysin O was also able to bind and form pores on archaeal membranes in the absence of cholesterol. In addition to improving the stability of the system, headgroup sugar moieties of archaeal lipids might also facilitate binding of certain pore-forming toxins and form functional pores.

*In vivo* stability and *in situ* blood stability of archaeosomes from *A. pernix* K1 were studied by Markelc et al.^32^ who intravenously injected calcein-filled archaeosomes (SUV; mean diameter: 200 nm) into female BALC/c mice with murine mammary adenocarcinoma TS/A cell line induced tumors. Circulation of archaeosomes in tumor blood vessels was determined using intravital microscopy carried out by a fluorescence stereomicroscope connected to a digital camera with image acquisition starting immediately after intravenous injection of the loaded archaeosomes. *In situ* blood stability was also studied by observing samples of the collected mouse blood mixed with loaded archaeosomes (1 to 10 ratio) at different times using a fluorescence microscope.

After intravenous injection into blood tumor vessels, steady flow of archaeosomes was observed within the first minute of injection with clustering of archaeosomes starting after 0.5 min. The detection of archaeosomes quickly decreased from 69 ± 5 vesicles per frame to 4 ± 1 at 30 min after injection, while the median diameter of circulating archaeosomes significantly increased from ∼1200 nm right after injection to ∼1900 nm at 3 min and to ∼2200 at 6 min. Extravasation of archaeosomes into the tumor tissue was not detected at any time.

**Figure 8:**
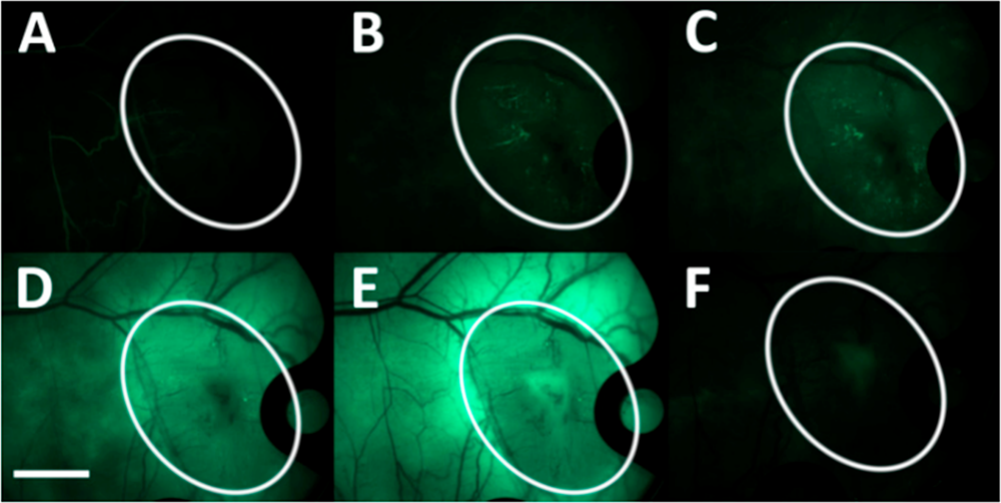
Intravital microscopy micrographs of the short- and long-term presence of C_25,25_ archaeosomes in normal and tumor blood vessels. Images were taken with a fluorescence microscope at different times (A – 5 s, B – 1 min, C – 2 min, D – 6 min, E – 30 min, F – 24 h) after intravenous injection of archaeosomes. Archaeosomes loaded with calcein are visible as bright green dots. Tumors are circled in white. Scale bar: 2 mm. Reprinted with permission from Markelc, B.; Napotnik, T. B.; Ota, A.; Cemazar, M.; Ulrih, N. P.; Miklavcic, D.; Sersa, G. Archaeosomes Prepared from *Aeropyrum Pernix* K1 Lipids as an *In Vivo* Targeted Delivery System for Subsequent Nanosecond Electroporation. In sixth European Conference of the International Federation for Medical and Biological Engineering, Eds.; Lacković, I., Vasic, D.; IFMBE Proceedings; Springer International Publishing: Cham, 2015; Vol. 45, pp 561–564. 10.1007/978-3-319-11128-5_140. Copyright 2015 Springer Nature.^32^

The circulating archaeosomes were immediately (5 s post intravenous injection) visible in the arteries (Figure 8A); however, at 1 min they predominantly localized into the tumor blood vessels (Figure 8B,C). The number of archaeosomes was significantly decreased at 6 min accompanied by an increase in fluorescence intensity in the surrounding tissue (Figure 8D,E), corroborating previous results. At 24 h, blood vessels were completely devoid of any archaeosomes or their aggregates (Figure 8F). There was also no change in weight or behavior of the studied mice, indicating that archaeosomes did not infer acutely toxic effects.

During *in situ* studies of archaeosomal stability in mouse blood, the authors attributed the observed flashes of increased fluorescence intensity to the effects of quick degradation and rapid bursting of archaeosomes (observed already within the first minute). The number of archaeosomes then steadily dropped, and at 30 min several aggregation clusters of archaeosomes were formed. In contrast, control archaeosomes that were only mixed with the buffer did not exhibit this behavior and remained intact within the same time frame.^32^

The low stability of archaeosomes *in vivo* could be a consequence of macrophage and dendritic cell activations which can also eliminate them. It should be noted that the mean diameter of archaeosomes used by Markelc et al.^32^ was 200 nm, which is relatively large for liposomal drug delivery systems (mean diameter is usually around 100 nm) and could impact their stability in biological systems. Additionally, the resolution of their system was limited to >400 nm. Smaller vesicles might also improve the delivery of drugs by extravasation of archaeosomes into the tumor tissue, which was not observed at the tested size range.

## Conclusion and Future Outlook

This review shows how and why the lipid membranes of extremophilic archaea have unique structural features that enable their biological functions in their respective environments. Studies of archaeal lipids must be conducted for each individual archaeon because their lipids vary widely between species. A deep understanding of the physicochemical properties of archaeal lipids gives us insight into how they thrive under extreme environmental conditions where life was long thought to be absent. As research on liposome-based drug delivery systems gained momentum, researchers focused on exploiting these properties of archaeal lipids

Promising properties such as adjuvant activity^25^ and physicochemical stability^8^ have made archaeal lipids potential drug delivery systems. However, their limited stability in biological systems (in contrast to adjuvants), nonstandard procedures and general difficulties in lipid isolation, and complex technical processes to maintain archaeal growth still require efficient solutions. Many studies have shown that combining archaeal lipids with conventional ester lipids is a promising strategy. These mixed vesicles still exhibit the highlighted properties of archaeal lipids, contained here in smaller ratios. This area of research is expected to expand in the future, particularly with respect to understanding the role of multicomponent archaeosomal membrane systems, multilayer integrated archaeosomes, and novel archaeosomal architectures. Archaeosomes derived from diether lipids of *A. pernix* have been shown to be comparable to archaeosomes derived from tetraether lipids, exhibiting good long-term stability at high temperatures and pH ranging from 4.0 to 12.0^13^ and low *in vitro* cytotoxicity.^24^ However, the *in vivo* circulation time of archaeosomes was unfavorable at <10 min. It should be noted that all the above studies on biological stability were performed with pure archaeosomes. Other possibilities for prospective studies include the addition of PEG-linked lipids and the subsequent formation of mixed vesicles, which might have better *in vivo* stability compared with the pure formulation, due to the lower proportion of archaeal lipid and the additional *in vivo* stabilization effect of certain lipids, especially those already used in clinical applications (e.g., PEG lipids, binary sphingomyelin/cholesterol formulations).^31^ In addition, archaeosomes derived from diether lipids of *A. pernix* could be used as a system for controlled drug delivery, as it has been shown that intact archaeosomes that are endocytosed release their charge on demand by nanosecond electroporation,^28^ provided they have sufficient biological stability to reach the target site.

Ultimately, mixed formulations have unpredictable behavior and therefore require thorough characterization studies aimed at finding optimal lipid ratios for potential applications that exhibit the best physicochemical stability and stability in biological systems. In addition to improved physicochemical stability, the addition of archaeal lipids to conventional lipids showed other advantages, such as easier preparation of vesicles due to the lack of transition from gel-like to a liquid-crystalline phase. Archaeal lipids continue to be an interesting area of research, offering favorable and promising properties for drug delivery systems. This review focuses on lipids from *A. pernix* that have been thoroughly characterized and shown to be potentially interesting liposomal drug delivery systems. In addition, the cultivation of *A. pernix* may be economically viable under various conditions due to the relatively simple biotechnological process and the physicochemical stability of the lipid composition.
